# Comparison of 3D and 2D gamma passing rate criteria for detection sensitivity to IMRT delivery errors

**DOI:** 10.1002/acm2.12389

**Published:** 2018-06-15

**Authors:** Dandan Zhang, Bin Wang, Guangshun Zhang, Charlie Ma, Xiaowu Deng

**Affiliations:** ^1^ Department of Radiation Oncology State Key Laboratory of Oncology in South China Collaborative Innovation Center for Cancer Medicine Sun Yat‐sen University Cancer Center Guangzhou China; ^2^ School of Physics Sun Yat‐sen University Guangzhou China; ^3^ Department of Radiation Oncology Fox Chase Cancer Center Philadelphia PA USA

**Keywords:** 2D and 3D gamma analysis, prostate IMRT QA, detection sensitivity, delivery errors

## Abstract

This study compared three‐dimensional (3D) and two‐dimensional (2D) percentage gamma passing rates (%GPs) for detection sensitivity to IMRT delivery errors and investigated the correlation between two kinds of %GP. Eleven prostate IMRT cases were selected, and errors in multileaf collimator (MLC) bank sag, MLC leaf traveling, and machine output were simulated by recalculating the dose distributions in patients. 2D doses were extracted from the 3D doses at the isocenter position. The 3D and 2D %GPs with different gamma criteria were then obtained by comparing the recalculated and original doses in specific regions of interest (ROI), such as the whole body, the planning target volume (PTV), the bladder, and the rectum. The sensitivities to simulated errors of the two types of %GP were compared, and the correlation between the 2D and 3D %GPs for different ROIs were analyzed. For the whole‐body evaluation, both the 2D and 3D %GPs with the 3%/3 mm criterion were above 90% for all tested MLC errors and for MU deviations up to 4%, and the 3D %GP was higher than the 2D %GP. In organ‐specific evaluations, the PTV‐specific 2D and 3D %GP gradients were −4.70% and −5.14% per millimeter of the MLC traveling error, and −17.79% and −20.50% per percentage of MU error, respectively. However, a stricter criterion (2%/1 mm) was needed to detect the tested MLC sag error. The Pearson correlation analysis showed a significant strong correlation (*r* > 0.8 and *P* < 0.001) between the 2D and 3D %GPs in the whole body and PTV‐specific gamma evaluations. The whole‐body %GP with the 3%/3 mm criterion was inadequate to detect the tested MLC and MU errors, and a stricter criterion may be needed. The PTV‐specific gamma evaluation helped to improve the sensitivity of the error detection, especially using the 3D GP%.

## 1 INTRODUCTION

Both the planning and delivery of intensity‐modulated radiation therapy (IMRT) are highly complex processes that require a comprehensive quality assurance (QA) procedure for routine IMRT plan verification.^1^, ^2^ Currently, IMRT QA is mostly performed by applying a patient‐specific treatment plan to a phantom, measuring the two‐dimensional (2D) planar dose distribution in the phantom, and comparing the measured and calculated phantom dose distributions. QA measurements are commonly taken with detector arrays consisting of either ion chambers or diodes. However, because of the lack of information regarding correlations between phantom dosimetry and anatomical dose distributions, including the volumetric dose differences between the targets and organs at risk (OARs), radiotherapy practice demands 3D dose verification based on actual patient anatomies.^3^ Several commercial 3D QA systems, such as 3DVH (Sun Nuclear Corporation, Melbourne, FL, USA), Compass (IBA Dosimetry, Inc., Memphis, TN, USA), DosimetryCheck (MathResolutions LLC, Columbia, MD, USA), and Mobius FX solution (Mobius Medical Systems, Houston, TX, USA), are capable of 3D dose reconstruction for pretreatment IMRT QA based on measurements. To quantitate the results of the IMRT QA measurements, gamma analysis based on the absolute dose difference (DD) and relative distance‐to‐agreement (DTA) between the measurement and the IMRT plan are widely used, both in 2D and 3D QA evaluations.^1^, ^4^

Previous studies have assessed the merits and limitations of different QA systems in terms of their compatibility with the gamma analysis methods and their capability to detect different IMRT delivery errors. Rangel et al.^5^ and Nelms et al.^6^ deliberately introduced systematic multileaf collimator (MLC) offset to the treatment beams and found that the 2D gamma analysis was insufficiently sensitive to detect some types of MLC misplacements and that planar IMRT QA passing rates did not predict clinically relevant patient dose errors. Pulliam et al.^7^ reported the findings of their phantom study: that the 3D gamma index produced better agreement than the corresponding 2D analysis with different algorithms. However, the responses of 3D and 2D gamma passing rates (%GPs) to different IMRT delivery errors have not yet been investigated thoroughly for individual structures in patients.

Therefore, this work applied the gradient technique and statistical methods to compare the 3D and 2D %GPs for different individual structures, to analyze the %GP responses to three different types of delivery error, and to investigate possible correlations between the two types of %GP.

## 2 MATERIALS AND METHODS

### 2.A Patient plans

Eleven IMRT plans for prostate treatment were randomly selected from clinical treatment cases. All patient plans were inversely optimized in the treatment planning system (TPS, Eclipse V11.0, Varian Medical Systems, Palo Alto, CA, USA) and calculated with a 2.5 mm × 2.5 mm × 2.5 mm dose grid using the anisotropic analytical algorithm (AAA). The plan isocenters were positioned at the PTV centroids. The plans consisted of eight or nine gantry angles and were delivered with the sliding window (SW) technique using 6‐MV photon beams from a linear accelerator (Trilogy, Varian Medical Systems, Palo Alto, CA, USA). The dose distributions of all 11 plans were exported in DICOM format as the error‐free dose references for each plan.

This study was reviewed and approved by the Institutional Review Board of Sun Yat‐sen University Cancer Center (IRB No. YB2017‐042).

### 2.B Introducing delivery errors into treatment plans

**Figure 1:**
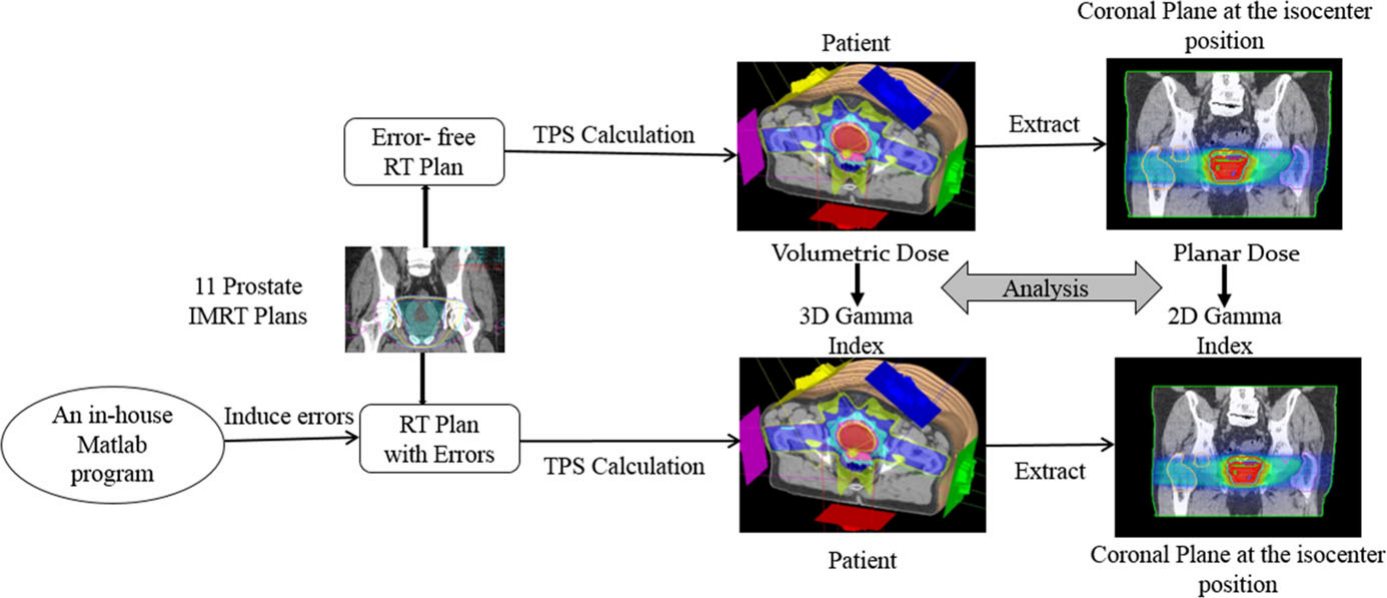
Experimental design. Simulated errors were introduced to error‐free RT plans, and the 3D and 2D %GPs (in the coronal plane) were then calculated to compare sensitivities to different errors and their correlations.

An in‐house MATLAB program was developed to insert three different types of delivery errors into the clinical plans, following the techniques described by Zhen et al.^8^ and Oliver et al.^9^ A brief description of the experimental flow is shown in Fig. 1. The three types of errors that were created were MLC bank sag errors, MLC traveling errors, and delivered machine output errors, all of which are common errors that may occur during treatment.

#### 2.B.1 MLC sag errors

Because IMRT is implemented by beams at varied gantry angles, the MLC bank sag error deserves significant attention. When the MLC control mechanism is relaxed, MLC sag errors may occur as a result of the gravity effect, with the most sag deviation occurring in MLC bank positions at gantry angles of 90° or 270° and no deviation at 0° or 180°. This type of error varies with the gantry rotation. We used a sinusoidal transform to simulate such a sag error, as reported by Carver et al.^10^

(1) $$MLC_{\mathrm{mod}}=MLC_{\mathrm{orig}}+Asin\left(\alpha \right)$$

The gantry angle *α* can be extracted from the plan's DICOM RT file for each corresponding control point. “A” is the maximum amplitude of the MLC leaf position change when the beam is horizontal; in this study, “A” was set to 1, 1.5, 2, and 3 mm.

#### 2.B.2 MLC leaf traveling errors

Another common type of error is the MLC leaf traveling error, which randomly occurs in some leaves and can be identified by the MLC picket fence (PF) test. Antypas et al.^11^ observed some individual MLC dispositions of greater than 1 mm by applying the PF test with an EBT2 film. In dynamic MLC delivery, numerous factors may cause inaccuracies in the individual leaf positions, including leaf motor problems and count losses by the primary encoders.^12^ In this study, we designed a model to simulate the traveling error of the two individual MLC leaves at the center position, assuming that these leaves are most likely to affect the target dose accuracy.

(2) $$MLC{\left(30,31\right)}_{\mathrm{mod}}=MLC{\left(30,31\right)}_{\mathrm{orig}}-B$$

The MLCs (30, 31) are the two central MLC leaves in the negative x‐direction (using the IEC 1217 coordinate system); B is the leaf positional error, which was set to 1.5, 2, 3, 4, and 5 mm in this work.

#### 2.B.3 Delivered MU errors

A third common type of error is the accelerator output error. This could be introduced by changing the number of MUs for each beam in the plan's DICOM RT files.

(3) $$MU_{\mathrm{mod}}=MU_{\mathrm{orig}}\left(1+C\right)$$

Here, C is the percentage linac output error, which was set to 3, 3.25, 3.5, 4, and 5%, respectively, in this study.

Based on the above assumptions, a virtual “RT delivery with errors,” shown in Fig. 1, was simulated by modifying each control point within the DICOM RT files of the selected IMRT plan, resulting in 154 modified plans. The dose distributions in patients were obtained by recalculating these plans using the TPS after modifying them with the virtual delivery errors. The planar and volumetric doses of both the error‐free plans and the plans with virtual errors were exported in DICOM format from the TPS onto 1‐mm planar and cubic grids.

### 2.C Gamma analysis and comparison

#### 2.C.1 Gamma evaluation

By comparing the recalculated and original plan doses, both the 2D and 3D %GPs were computed with the absolute dose difference normalized to the global maximum dose and the relative DTA in both 2D and 3D way. The 3D %GP was calculated by extending the 2D %GP calculation reported by Low et al.^3^ to a third dimension. The 3DVH software V2.2.0 (Sun Nuclear Corporation, Melbourne, FL, USA) was used to compute the 3D %GP, and the Patient software V6.2.3 (Sun Nuclear Corporation, Melbourne, FL, USA) was applied for the 2D gamma analysis. The 3D and 2D %GPs for the whole body (CT‐scanned area), PTV, and OARs were calculated separately and then compared.

**Figure 2:**
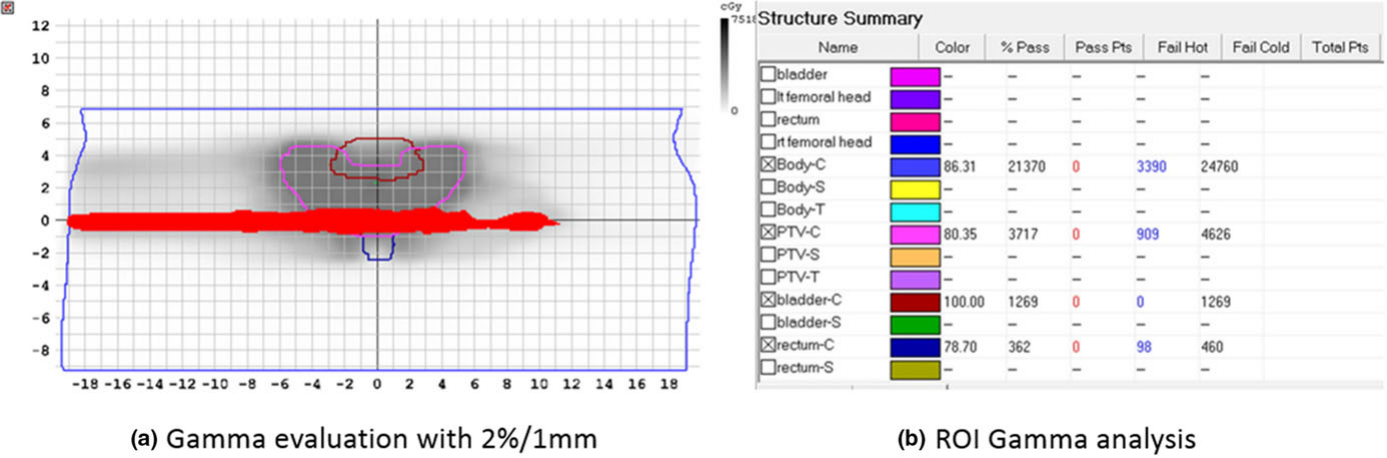
An example of the 2D gamma analysis result with the criterion of 2%/1 mm for a 2‐mm MLC leaf misalignment. (a) 2D gamma map: ROIs, including the PTV and OARs in the coronal slice at the isocenter were transferred from the TPS plan and used to define the region for planar gamma analysis; the points where the gamma evaluation failed are shown in red. (b) Summary of the 2D gamma analyses for different structures.

For the 2D gamma analyses of the individual structures, the 2D region of interest (ROI) contours were extracted from the corresponding coronal plane of the 3D structure dataset in the TPS and transferred to the Patient software. The relative lower dose threshold (TH) value was set to 10%. The OARs investigated in this study were the bladder and rectum, which are adjacent to the PTV in the prostate IMRT (Fig. 2).

In clinical practice, the 2D %GP criterion of 3%/3 mm (DD%/DTA mm) has been commonly recommended and routinely applied for IMRT QA.^1^ Nevertheless, a 3% dose output tolerance and a 2‐mm mechanical accuracy are recommended for daily QA using conventional IMRT machines, and a stricter tolerance is recommended for stereotactic body radiation (SBRT) treatment.^13^ Therefore, the 3%/2 mm criterion is usually used as a more restrictive %GP criterion, and the gamma criterion of 2%/1 mm has been recommended for detecting MLC shift misalignments in SBRT QA.^14^ In this study, three different criteria of 2%/1 mm, 3%/2 mm, and 3%/3 mm were applied for the gamma evaluations in both the 2D and 3D %GP calculations. Two‐tailed paired‐sample *t* tests was used to compare the 2D and 3D %GPs. Statistical significance was defined by a *P* value <0.05 with the SPSS 18.0 software (SPSS Inc., Chicago, IL, USA).

#### 2.C.2 Gradient analysis of %GP per unit error magnitude

To compare the sensitivity of different %GPs to simulated errors quantitatively, a %GP gradient technique was adopted in this study. Liang et al.^15^ used this approach to analyze sensitivity to machine errors during volumetric‐modulated arc therapy (VMAT) delivery for three commercial QA systems.

The average %GPs and corresponding error magnitudes were linearly regressed through least‐squares fitting, and the slope of the linear fitting indicated the average decrease in %GP per unit of simulated error. Higher slopes represented %GPs that were more sensitive to detection error. To evaluate specific %GP gradients for each type of error, the default gamma criterion (DD%/DTA mm) was set to 3%/3 mm.

#### 2.C.3 Correlation analysis between the 2D and 3D %GP

The correlation between the 2D and 3D %GPs was statistically evaluated by analyzing the Pearson correlation coefficient using the SPSS software V18.0 and pair plots. A Pearson's *r* value greater than 0.8 in conjunction with a *P* value of less than 0.05 in the significance test was considered to indicate a strong correlation.

## 3 RESULTS

### 3.A 2D vs 3D %GP analyses

The 2D and 3D %GPs were compared with three different criteria of 2%/1 mm, 3%/2 mm, and 3%/3 mm. The average results of the %GPs for the body, PTV, and OARs for the three types of error are shown in Tables 1, 2, 3. The results were summarized as follows.

**Table 1:** Comparison of average 2D %GP and 3D %GP with MLC sag errors

%GP Area	Simulated error	2%/1 mm			3%2 mm			3%/3 mm		
		2D	3D	*P* value	2D	3D	*P* value	2D	3D	*P* value
Body	1 mm*^A^	94.19	96.53	0.145	99.31	99.88	***0.022***	99.80	99.97	***0.037***
	1.5 mm*^A^	87.02	86.59	0.85	96.98	99.59	***0.013***	98.55	99.93	***0.013***
	2 mm*^A^	79.62	79.82	0.932	93.73	96.21	0.151	96.29	99.87	***0.007***
	3 mm*^A^	66.72	72.05	0.081	85.45	90.53	0.068	90.05	98.11	***0.004***
PTV	1 mm*^A^	98.81	96.55	***0.032***	100.00	99.47	0.286	100.00	99.84	0.341
	1.5 mm*^A^	97.66	92.05	***0.001***	99.50	98.82	0.385	99.98	99.70	0.308
	2 mm*^A^	95.46	88.89	***0.002***	99.19	95.94	***0.002***	99.46	99.59	0.831
	3 mm*^A^	89.23	83.78	***0.046***	97.68	93.69	***0.01***	98.72	97.64	0.207
Bladder	1 mm*^A^	96.96	93.92	0.332	100.00	99.99	0.343	100.00	100.00	NA
	1.5 mm*^A^	90.14	81.58	0.167	98.97	99.86	0.416	99.53	100.00	0.343
	2 mm*^A^	82.79	73.11	0.253	96.90	90.89	0.115	98.12	100.00	0.333
	3 mm*^A^	70.98	64.27	0.58	90.08	88.01	0.748	96.12	96.97	0.78
Rectum	1 mm*^A^	45.07	84.87	0.262	72.68	99.53	0.307	87.73	99.90	0.423
	1.5 mm*^A^	41.09	53.26	0.622	50.65	97.48	0.234	71.88	99.73	0.299
	2 mm*^A^	40.51	44.58	0.822	46.01	82.80	0.275	53.86	99.41	0.236
	3 mm*^A^	40.43	41.65	0.824	42.32	68.34	0.767	45.43	88.53	0.592

*A refers to the variable in eq. (1).

**Bold italic font highlights *P* values ≤ 0.05.

**Table 2:** Comparison of average 2D %GP and 3D %GP with MLC traveling errors

%GP Area	Simulated Error	2%/1 mm			3%2 mm			3%/3 mm		
		2D	3D	*P* value	2D	3D	*P* value	2D	3D	*P* value
Body	1.5 mm*^B^	92.71	94.95	***0.015***	97.15	98.06	0.088	98.27	98.80	0.104
	2 mm*^B^	89.38	92.31	***0.008***	94.36	96.02	***0.032***	96.09	97.17	0.058
	3 mm*^B^	86.87	90.99	***0.015***	92.68	94.02	0.225	94.40	95.25	0.302
	4 mm*^B^	84.87	88.25	***0.003***	89.18	92.33	***0.010***	91.37	93.45	0.099
	5 mm*^B^	82.65	85.24	***0.009***	87.14	89.54	0.092	89.30	90.61	0.060
PTV	1.5 mm*^B^	80.66	80.48	0.921	88.87	85.87	***0.04***	91.56	87.34	***0.006***
	2 mm*^B^	75.96	76.15	0.924	83.68	81.58	0.123	86.38	82.06	***0.002***
	3 mm*^B^	72.56	72.86	0.879	80.04	78.63	0.309	82.78	78.99	***0.009***
	4 mm*^B^	69.82	70.23	0.828	77.41	76.23	0.447	80.29	76.66	***0.024***
	5 mm*^B^	65.30	65.85	0.784	73.39	72.33	0.493	75.92	72.84	***0.046***

*B refers to the variable in eq. (2).

**Bold italic font highlights *P* values ≤ 0.05.

**Table 3:** Comparison of average 2D %GP and 3D %GP with MU errors

%GP area	Simulated error	2%/1 mm			3%2 mm			3%/3 mm		
		2D	3D	*P* value	2D	3D	*P* value	2D	3D	*P* value
Body	3%*^C^	82.70	93.05	***0***	98.05	99.63	0.109	98.71	99.68	0.182
	3.25%*^C^	80.68	91.95	***0***	92.83	97.42	***0***	95.38	97.61	***0.015***
	3.50%*^C^	78.28	90.65	***0***	88.85	95.41	***0***	91.05	95.83	***0***
	4%*^C^	73.19	87.50	***0***	87.90	95.07	***0***	89.26	95.63	***0***
	5%*^C^	60.78	77.75	***0***	85.73	92.85	***0***	87.82	94.12	***0***
PTV	3%*^C^	3.29	2.43	0.302	80.55	91.55	0.292	86.98	92.11	0.684
	3.25%*^C^	2.76	2.01	0.36	46.30	48.81	0.626	65.38	50.89	***0.022***
	3.50%*^C^	2.28	1.66	0.399	19.26	8.09	***0***	34.57	12.92	***0***
	4%*^C^	1.57	1.12	0.436	13.38	5.79	***0.001***	21.97	9.97	***0***
	5%*^C^	0.69	0.55	0.68	8.42	3.84	***0.005***	16.24	6.75	***0***
Bladder	3%*^C^	52.35	81.28	***0.020***	95.16	99.51	0.299	97.63	99.62	0.439
	3.25%*^C^	50.90	79.56	***0.019***	85.46	93.94	0.099	92.37	94.53	0.534
	3.50%*^C^	49.93	76.89	***0.024***	66.52	87.29	0.064	75.52	89.19	0.13
	4%*^C^	46.77	70.07	***0.034***	62.67	86.01	***0.043***	68.67	88.29	0.08
	5%*^C^	33.31	53.18	***0.037***	58.07	82.99	***0.039***	64.84	86.45	0.062
Rectum	3%*^C^	91.52	89.56	0.919	100.00	99.47	0.423	100.00	99.62	0.423
	3.25%*^C^	91.01	89.04	0.942	97.39	96.68	0.974	98.62	97.20	0.788
	3.50%*^C^	90.06	88.41	0.985	96.67	92.89	0.357	97.68	94.36	0.240
	4%*^C^	87.27	87.22	0.857	94.06	92.22	0.775	96.88	93.74	0.371
	5%*^C^	76.09	82.27	0.537	92.97	91.15	0.813	95.43	92.80	0.566

*C refers to the different variable in the eq. (3).

**Bold italic font stands for *P* value ≤0.05.

#### 3.A.1 MLC bank sag error detection sensitivities

**Figure 3:**
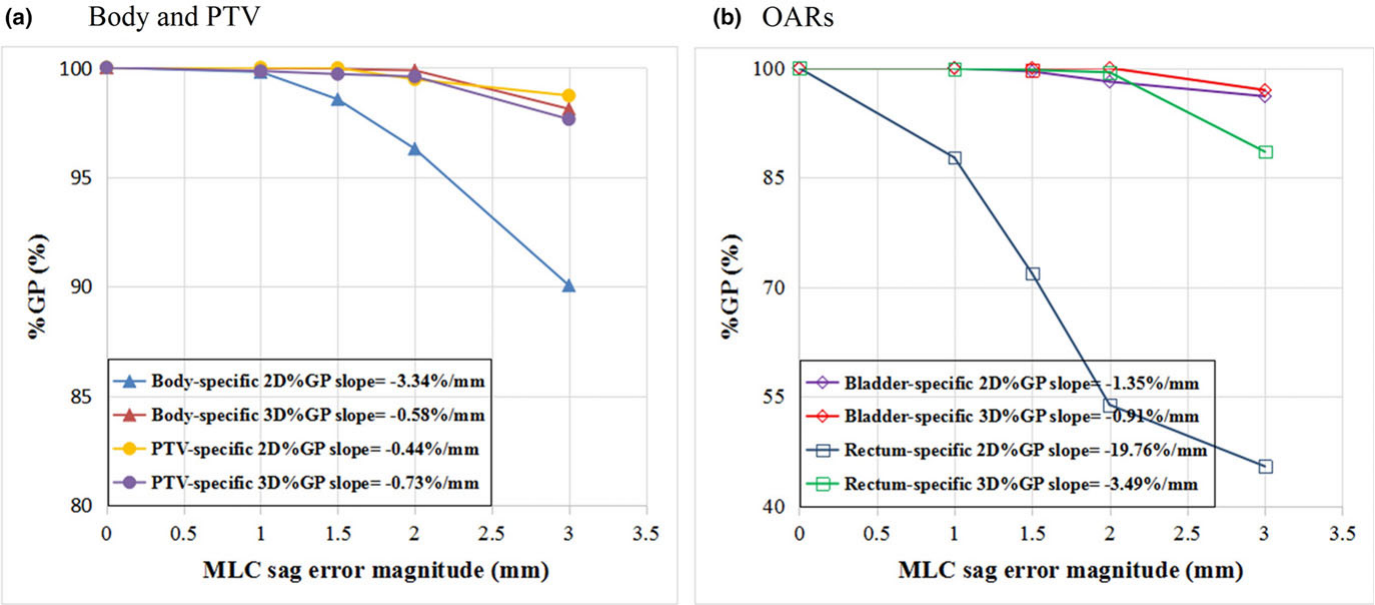
**MLC** **sag error sensitivity analysis using gradient technique. (a) Whole body and** **PTV** **. (b)** **OAR** **s.**

**Figure 4:**
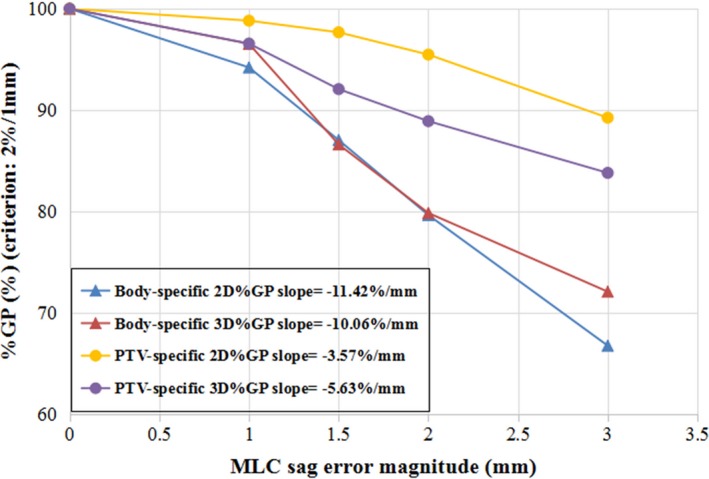
**%** **GP** **with 2%/1 mm criterion vs** **MLC** **sag error.**

With regard to the MLC bank sag errors, with the 3%/3 mm criterion, the 2D %GP was lower than the 3D %GP in the whole body (*P* < 0.05); however, no significant difference between the two was observed in any of the contoured structures (*P* > 0.05). With the stricter criterion of 2%/1 mm, all %GPs except the 2D %GP for the rectum were higher than or in the vicinity of approximately 90% when the MLC sag error was less the 1.5 mm, showing a relatively low sensitivity to the MLC sag error at this level. In particular, the 3D %GPs were all above 99% at the 3%/3 mm criterion in all the structures, even when the MLC sag error was as large as 2 mm. Figure 3 shows the average whole‐body %GP for a linear regression function of error magnitude. The gradient of the 2D %GP (−3.34%/mm) was steeper than that of the 3D %GP (−0.58%/mm) for the whole body area, indicating that the 2D %GP may be more sensitive to MLC sag error than the 3D %GP [Fig. 3(a)] for whole‐body gamma evaluations. Of all the structure‐specific evaluations, the rectum‐specific %GP had the largest absolute slope [Fig. 3(b)]. In contrast, when a stricter criterion of 2%/1 mm was used, the 3D %GP was significantly lower than the 2D %GP (*P* < 0.05), as shown in Table 1, and had a steeper gradient, as shown in Fig. 4, indicating that the PTV‐specific 3D %GPs with the stricter criteria were also more sensitive to the MLC sag error.

#### 3.A.2 MLC leaf traveling error detection sensitivities

**Figure 5:**
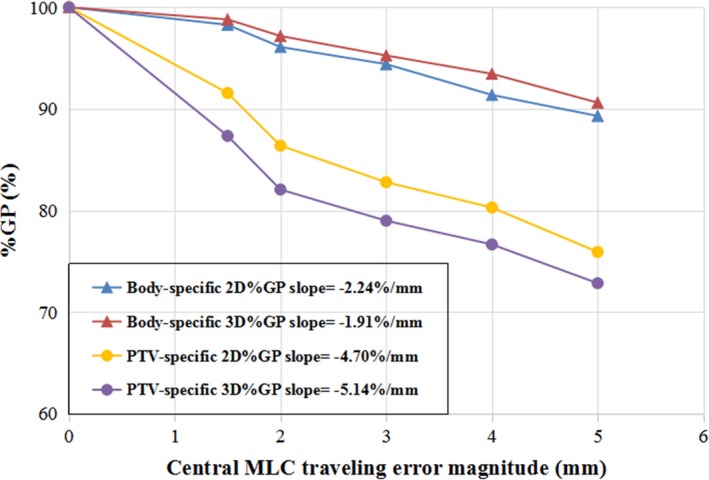
%GP vs MLC leaf traveling error.

Because the simulated MLC leaf traveling errors were introduced to only the two central leaves, which are unlikely to affect the dose accuracy in the OARs above or below the PTV level, the OAR‐specific %GPs were not assessed for this type of error. The results of the %GP evaluation for the whole body and the PTV‐specific region are shown in Table 2 and Fig. 5.

Both the 2D and 3D %GPs evaluated for the whole body with the 3%/3 mm criterion were insensitive to the simulated MLC traveling error in the two central leaves. The %GP maintained a relative high value above 90% for all the tested leaf traveling error levels, even when the error was up to 3 mm (sufficiently large to change the dose accuracy in the PTV). The 2D %GP was significantly lower (*P* < 0.05) than the 3D %GP with the criterion of 2%/1 mm in the evaluation of the whole body area, indicating that the 2D %GP with a stricter criterion may be more sensitive than the 3D %GP for detecting MLC leaf traveling errors in whole‐body evaluations. In the %GP evaluation for the PTV‐specific region, both the average 2D and 3D %GPs were lower than 90% for virtual MLC leaf traveling errors greater than 1.5 mm, and the %GPs decreased quite rapidly as larger MLC errors were introduced, showing better sensitivity to this type of error. However, within the PTV region, the 3D %GP with the 3%/3 mm criterion was lower than the 2D %GP for all tested error levels (*P* < 0.05), with an average 3D %GP of 87.34% vs an average 2D %GP of 91.56% for a 1.5‐mm MLC leaf traveling error.

As shown in Fig. 5, the %GP gradient analysis supported the above results with a larger absolute slope in the PTV‐specific %GP than that of the whole body and a steeper decline in the PTV‐specific 3D %GP than the PTV‐specific 2D %GP.

#### 3.A.3 MU error detection sensitivities

**Figure 6:**
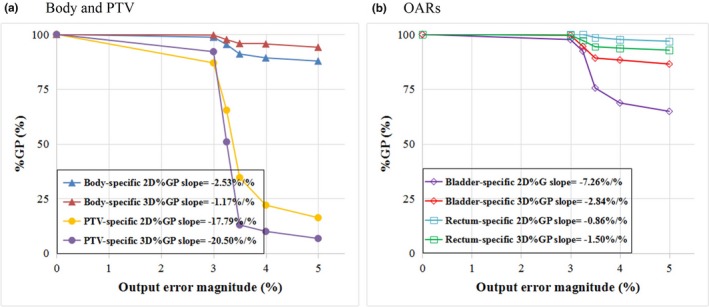
Sensitivity to output error. (a) Whole body and PTV. (b) OARs.

For different levels of MU errors, the PTV‐specific %GPs were very sensitive with criteria corresponding to the output error level in both the 2D and 3D evaluations. The %GP decreased considerably when the MU error surpassed the DD criterion of the gamma evaluation, and the 3D %GP declined more abruptly than the 2D %GP in the PTV‐specific evaluation. However, in the gamma evaluations for the whole body and the bladder, the %GP decreases were relatively gradual than that for the PTV, and the average 2D %GP was significantly lower (*P* < 0.05) or similar (*P* > 0.05) to that of the 3D evaluation in these areas. The results of the MU error testing are detailed in Table 3 and Fig. 6. For this type of error, the PTV‐specific 3D %GP had the largest absolute slope.

### 3.B Correlation between 2D %GP and 3D %GP

**Figure 7:**
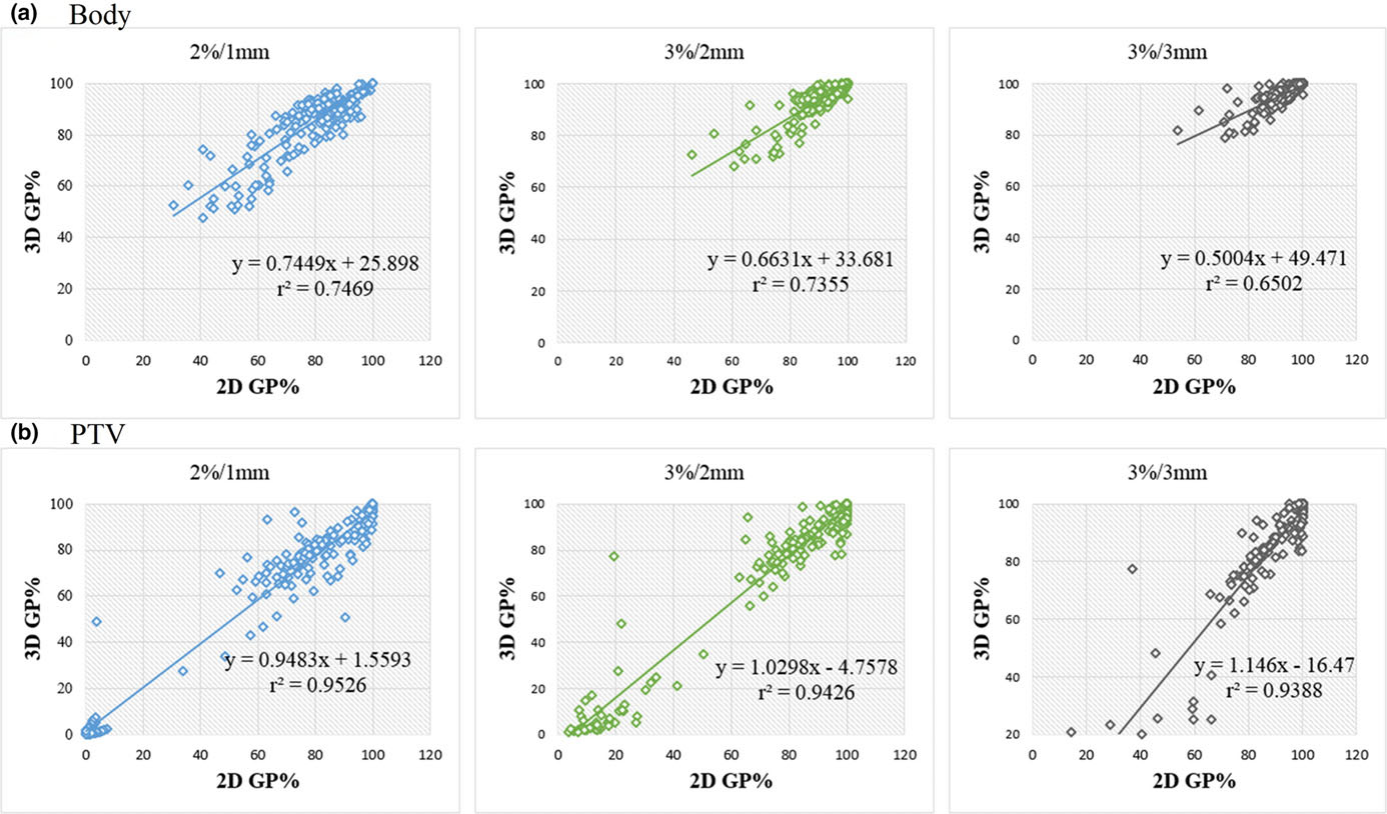
Scatter plot of 2D %GP vs 3D %GP for (a) the whole body and (b) the PTV with different errors and gamma criteria (2%/1 mm, 3%/2 mm, 3%/3 mm; 10% threshold cutoff).

Figure 7 illustrates the 2D %GP vs 3D %GP for all 154 modified prostate plans, with the different gamma evaluation criteria (2%/1 mm, 3%/2 mm, and 3%/3 mm). Table 4 presents the statistical results of the Pearson correlation, with the *P* values of the two‐tailed tests between the 2D and 3D %GPs for different structures.

**Table 4:** Pearson correlation coefficients with corresponding two‐tailed *P* values correlating 2D %GP to 3D %GP

DD/DTA	2%/1 mm		3%/2 mm		3%/3 mm	
	R	*P*	r	*P*	r	*P*
Body	0.864	<0.001	0.858	<0.001	0.806	<0.001
PTV	0.976	<0.001	0.971	<0.001	0.969	<0.001
Bladder	0.532	<0.001	0.463	<0.001	0.5	<0.001
Rectum	0.653	<0.001	0.566	<0.001	0.504	<0.001

The scatter plots show the linear relationship between the 2D and 3D %GPs for the whole body and the PTV region. Shown in the statistical analysis, a significant strong correlation existed between the 2D and 3D %GPs at all tested gamma evaluation criteria for both the whole body and the PTV region (*r* > 0.8 and *P* < 0.001). The Pearson's correlation coefficients of the 2D and 3D %GPs for the PTV were higher than those for the whole body, with *r* values (for different criteria) of 0.976 (2%/1 mm), 0.971 (3%/2 mm), and 0.969 (3%/3 mm) for the PTV vs 0.864 (2%/1 mm), 0.858 (3%/2 mm), and 0.806 (3%/3 mm) for the whole body. However, as shown in Table 4, the correlations between the 2D and 3D %GPs for the OARs were weaker than those for the whole body and the PTV. For the bladder and rectum, the correlations between the 2D and 3D %GPs at all the tested criteria were weak (*r* ≤ 0.8).

## 4 DISCUSSION

Although recent publications have questioned the utility of %GP because of the perceived lack of a strong relationship with clinical DVH metrics,^5^, ^16^, ^17^ no consensus has been reached regarding an acceptable replacement for the %GP metric in the field of IMRT QA. As reported in AAPM TG119, despite several weaknesses, gamma analysis remains a valuable and widely used method in clinical practice.^1^ This study aimed to remedy the %GP limitation by comparing the sensitivities of different %GPs to different delivery errors and investigating the relationship between the 2D and 3D %GPs to aid the selection of appropriate gamma analysis methods for detecting specific errors that may occur in clinical practice.

For the most commonly used whole‐body gamma analysis, the results of our study show that neither the 2D nor the 3D %GP was sufficiently sensitive to detect a sag error of 1.5 mm, although the 2D %GP appeared to be more sensitive than the 3D GP% when the sag error was worse. When the 3%/3 mm criterion was used, both the 2D and 3D %GPs were higher than 90% for sag errors up to 3 mm. This result agreed with those reported by Heilemann et al.,^18^ where the 2D coronal %GP with the 3%/3 mm criterion decreased only 4.3% in response to a 3‐mm MLC sag shift in a rapid‐arc delivery for prostate treatment, and a stricter criterion was needed to detect such a large MLC sag error.

Yan et al.^19^ simulated systematic and random MLC traveling errors of up to 2 mm and found that the 2D %GP of the whole body area was insufficient to identify random MLC errors of up to 2 mm, even when a stricter criterion of 2%/2 mm was used. Similarly, we found that both the 2D and 3D %GPs for the whole body responded only mildly to the tested leaf traveling errors. Nevertheless, the PTV‐specific %GP was sensitive to the tested traveling error of the two central MLC leaves because this error most affected the dose distribution in the PTV region. In this case, the 3D %GP for the PTV was significantly lower than the 2D %GP (*P* < 0.05); in addition, the 3D %GP showed a steeper gradient when the traveling error was less than 1.5 mm, showing that the 3D %GP of the PTV may be more sensitive than the 2D %GP for a small leaf traveling error in the central leaves.

For the MU error, our results indicated clear responses from both the 2D and 3D %GP responses to MU errors over the DD criterion. Although the 3D %GP declined even more quickly than the 2D %GP in the PTV for a small MU error, this difference became quite small when the MU error was bigger.

Pulliam et al.^7^ and Sun et al.^20^ observed that the average 3D %GP was approximately 3% higher than the 2D %GP in the whole measurement area, and they attributed this difference to the definition of the gamma value because the extra dimension used to search for matching results increased the number of tested pixel points in the 3D gamma analysis. The results of our study agree with the above literature, with a higher 3D %GP for all types of simulated error in the gamma evaluation of the whole body area. However, in the PTV‐specific gamma evaluation, the 3D %GPs were lower and had a steeper gradient than the 2D %GPs for the tested MLC errors. Wu et al.^21^ also compared the PTV‐specific 3D %GP with the 2D %GP for 56 IMRT cases and reported similar results (3D %GP of 96.93% vs 2D %GP of 98.62% on average). A possible reason for this result could be that, when an MLC error occurred, the DTA was affected more at the edge of the PTV where the dose gradient was sharper, and the 3D %GP was more sensitive than the 2D %GP for the DTA deviations.

Another issue addressed in our study is the correlation between the 2D and 3D %GPs. A significant strong correlation was observed between them for the PTV and whole‐body gamma evaluations (Pearson *r* > 0.8 and *P* < 0.05). Similar results were reported by Wu et al.^21^ in which they observed a significant statistical correlation between 3D and 2D global (body area) %GPs in their investigation of two IMRT QA methods.

## 5 CONCLUSIONS

In this work, we investigated the detection sensitivities to three typical delivery errors of 2D and 3D %GPs and their correlations. For the whole‐body gamma evaluation, both the 2D and 3D %GPs with the commonly used criterion of 3%/3 mm were inadequate to discover small MLC sag and leaf traveling errors. The PTV‐specific 3D %GP evaluations with the stricter criterion were more sensitive to detect these types of MLC errors. A corresponding dose difference criterion is needed to detect MU errors using %GP, and PTV‐specific analysis is more sensitive to this type of error compared to whole‐body assessment.

## AVAILABILITY OF DATA AND MATERIALS

All data in this study have been recorded at the Research Data Deposit website (RDD, https://www.researchdata.org.cn) for future reference (number RDDA2017000326) and are available upon request.

## CONFLICT OF INTEREST

No conflicts of interest.
